# Endoscope-Assisted or Skin-Approach Osteosynthesis of Mandibular Condylar Fracture—A Comparison

**DOI:** 10.3390/jfb16100382

**Published:** 2025-10-11

**Authors:** Paulina Agier, Dominik Szczeciński, Marcin Kozakiewicz

**Affiliations:** 1 Multispecialty Dental Clinic, 106/116 Kościuszko Av., 90-442 Lodz, Poland; paulinaagier2402@gmail.com; 2 Department of Maxillofacial Surgery, Medical University of Lodz, 251 Pomorska Str., 92-213 Lodz, Poland; dominik.szczecinski@umed.lodz.pl

**Keywords:** mandible, condyle, fracture, fixation, osteosynthesis, ORIF, endoscope, surgical, treatment, complications

## Abstract

Open reduction and internal fixation (ORIF) for mandibular condyle fractures remains a controversial and challenging issue, with the exception of basal and low-neck fractures. Currently, there is a consensus that fractures causing irreparable malocclusion or dislocation, when the fracture line runs through the base or lower neck of the condyle, require ORIF. Due to the different characteristics of fractures, various surgical approaches and their modifications are available. The use of a minimally invasive intraoral approach during endoscope-assisted procedures is considered safer for the facial nerve and provides good esthetic results without facial scarring. This study aimed to compare two surgical approaches—retromandibular and intraoral—to examine post-operative outcomes and to guide surgical decision-making in the treatment of simple fractures of the base and low-neck condylar process of the mandible. Forty-nine patients (thirteen female, thirty-six male) were analyzed: eighteen were treated with the intraoral approach, and thirty-one with the retromandibular approach. There were no statistical differences in the duration of surgery, but intraoperative blood loss was significantly lower in patients treated endoscopically compared with those treated with an extraoral approach. Post-operative facial nerve and TMJ function were comparable in both groups. The endoscope-treated patients were at a higher risk of fracture non-union, but these findings should be considered with connection with the small sample size. The intraoral approach is a valuable option for basal or low-neck fractures but demands significant surgical experience due to its technical complexity.

## 1. Introduction

Fractures of the mandibular condyle are an important issue in maxillofacial traumatology. They are known to significantly impact patients’ well-being and quality of life due to possible complications [1]. Nowadays, three different treatment options are used for this condition. The methods are as follows: non-surgical treatment; open rigid internal fixation (ORIF) via an extraoral approach; and endoscope-assisted surgery via an intraoral approach. In order to select the most suitable treatment method for a mandibular condyle fracture, surgeons must establish the proper diagnosis of the fracture type. It is important to make a detailed diagnosis, with awareness of the indications and contraindications of the chosen treatment method [2,3,4,5]. Attention should also be paid to the patient’s general condition, comorbidities, and social conditions [6,7,8,9]. Conservative methods are indicated for pediatric population, in intracapsular fractures, fractures without displacement, in older patients with multiple comorbidities, or when general anesthesia is contraindicated [6,9,10,11]. A review of the literature indicates that there are differences in the indications for ORIF treatment. However, the most commonly reported clinical indications are as follows: displacement of bone fragments > 30° and ramus height shortening of ≥2 mm, dislocations in the temporomandibular joints (TMJ), no direct contact between bone fragments, comminuted fractures, foreign body or hematoma in the TMJ, bilateral condylar fractures, and laceration of the external acoustic meatus [5,7,12,13,14]. The advantages of ORIF treatment are well known and widely discussed, but the risk of post-operative complications is also a serious issue [15]. It is worth noting that immediate post-operative facial nerve palsy may occur even in approximately 50% of patients [16,17,18]. Other possible adverse outcomes include salivary fistulas [19,20], malocclusion, TMJ disorders [21] and unsightly facial scarring [22,23,24,25,26], among others. Considering the range of possible complications after ORIF [27], many surgeons have sought out more effective and innovative techniques for treating mandibular condylar fractures.

Nowadays, classical open procedures are increasingly being replaced by endoscope-assisted procedures in many fields of surgery. The same trend toward minimally invasive surgery is visible in maxillofacial surgery, where there is growing interest in using endoscopes for orthognathic surgery, sinus surgery, surgery for post-traumatic injuries, salivary gland surgery and TMJ arthroplasty [28,29,30]. An increasing number of centers worldwide are adopting endoscopic technique to treat condylar process fractures by using the intraoral approach [4,31,32,33].

The first mention of pioneering mandibular condylar process surgery using an endoscope dates back to 1998, when two teams independently published the results of their pioneering work in this field Schmelzeisen et al. [34] demonstrated anatomical results in seven condylar fractures treated with endoscope assistance, with only one case of failure. A more comprehensive report was presented by Lee et al. [35], who described the results of endoscopic treatment of 22 fractures. They highlighted successful restoration of TMJ function and better esthetic results than with open treatment.

Over the past few decades, endoscopic surgical techniques have been refined and become more widely practiced. There are now many reports in the literature detailing the indications for endoscopic treatment and its advantages. The current literature suggests considering endoscope-assisted treatment for patients with condylar base or low-neck fractures without dislocation or with small dislocations of bone fragments, as long as the fragments are still in contact and there are no TMJ dislocations. Moreover, the conditions of injury should enable the application of fixing material using an intraoral approach. However, it is important to emphasize that this technique demands surgical experience, making the learning curve both necessary and challenging [18,36,37,38]. A substantial number of studies have demonstrated that endoscope-assisted treatment is associated with a reduced risk of complications affecting the facial nerve when compared with ORIF treatment. This approach is also commonly linked to a reduced incidence of post -operative complications, including a less traumatic tissue handling, reduced post-surgical edema and pain, shorter time of patient’s recovery, and lower rate of indications for reoperation [18,39,40,41,42].

The aim of this study was to evaluate the risk factors and post-operative complications associated with traditional ORIF versus endoscope-assisted ORIF, and to present data to support the choice of surgical approach in the treatment of simple fractures of the base and low-neck region of the condylar process of the mandible.

## 2. Materials and Methods

This study was a retrospective analysis of medical records. Retrospective clinical data of patients treated between 2021 and 2023 at the Department of Maxillofacial Surgery, Medical University of Lodz were extracted from the hospital’s electronic database using ICD codes. Fractures of the mandibular condylar region were evaluated.

Criteria of inclusion were: adult patients, basal fracture, low-neck fracture, radiological examination, full medical records, patients attending the follow-up appointments. The following criteria were applied for exclusion: pediatric patients, high-neck fracture, mandible head fracture, incomplete medical records, a history of cancer, a metabolic bone disease, a rheumatic diseases, diabetes and neuromuscular disorders, lack of follow-up appointments.

The observational study was reported in accordance with the Strengthening the Reporting of Observational Studies in Epidemiology (STROBE) guidelines [43]. As this was a retrospective study, no a priori sample size calculation was performed. Instead, all available cases that fulfilled the predefined inclusion criteria were included in the analysis. This approach ensured comprehensive utilization of the accessible dataset while minimizing selection bias.

A total of 182 medical records of patients treated for mandibular condylar process fractures were reviewed. After exclusion of 133 records that did not meet the inclusion criteria, 49 cases were included in the final analysis. All 49 mandibular condylar fractures were treated by two specialist surgeons, each serving as the primary operator: one performed endoscope-assisted procedures via the intraoral approach, while the other exclusively applied the retromandibular approach. The follow-up period was 6 months.

The study collected data on patient demographics (age, gender, place of residence, body mass index [BMI]) and injury characteristics (type of condylar fracture, number of injured condyles, cause of injury). Preoperative blood tests included hematocrit level. Surgical data comprised duration of surgery and type of fixation. Post-operative assessments included hematocrit, C-reactive protein (CRP), Helkimo Index (0 = no dysfunction; I = mild; II = moderate; III = severe [21,44]) at 6 months, and House–Brackmann scale (1 = full nerve function; 6 = total dysfunction [45]) measured immediately after surgery and at 6 months. Data on post-operative complications and reoperations were also recorded.

Due to wide variability, in general, health data, it was not possible to determine the optimal sample size. The available budget allowed full analysis of 49 patients. Therefore, the case series was continued until treatment was completed in 31 consecutive patients in the retromandibular approach group and 18 in the intraoral approach group.

During the diagnostic process, the type of mandibular condylar fracture was determined by computed tomography (CT) using RadiAnt DICOM Viewer 2024.1 (https://www.radiantviewer.com/en accessed on 10 September 2025). Fractures were classified according to Neff’s and Kozakiewicz’s classifications [46,47]. Patients were divided into six diagnostic categories: head type A, head type B, head type C, high-neck, low-neck, and basal (only basal and low-neck fractures were included in this study). These assessments facilitated selection of the most appropriate treatment and surgical approach. Prior to surgery, venous blood tests were performed as part of the pre-operative evaluation.

**Figure 1:**
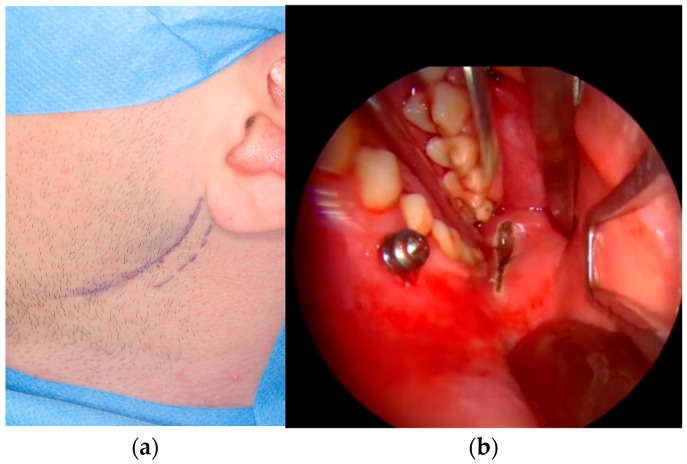
Visualization of the initial steps of the surgical approaches: orientation lines for the retromandibular approach (**a**) and incision for the intraoral approach (**b**).

**Figure 2:**
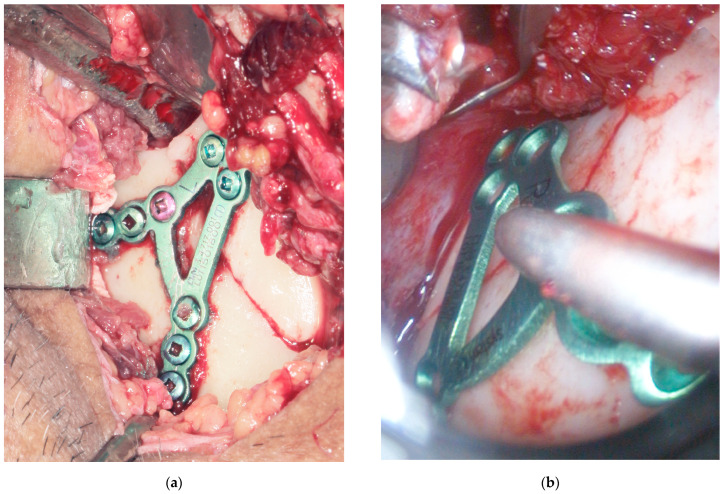
Example of ORIF via retromandibular approach contrary to intraoral approach with endoscope assistance: (**a**) retromadibular transparotid approach and osteosynthesis of basal fracture of mandibular condyle. The salivary gland visible in the upper right part; (**b**) intraoral approach with endoscope assistance. All dissection was performed below the masseter.

The surgical procedure was performed under general anesthesia with nasal intubation, without intramaxillary fixation. Antibiotic prophylaxis was administered. For patients treated with an extraoral approach, the retromandibular transparotid approach was used. Prior to the skin incision, the operative field was disinfected with a non-alcoholic antiseptic solution. Orientation lines were drawn on the skin (Figure 1a), and local infiltration with a vasoconstrictor (adrenaline 1:200,000–1:100,000) was performed. After approximately 15 min, once the vasoconstrictive effect was achieved, a skin incision was made. The incision was performed perpendicular to the surface, approximately 1 cm behind the posterior border of the mandibular ramus, extending from the earlobe to the region of the mandibular angle. The subcutaneous tissue was bluntly dissected anteriorly, followed by incision of the periauricular fascia. The parotid gland parenchyma was then divided through its superficial lobe. At this stage, the operative field was thoroughly irrigated with tranexamic acid to distinguish the branches of the facial nerve from the salivary gland ducts. Branches of the facial nerve that were not encountered were not actively sought, in order to minimize the risk of facial nerve injury in inchemic mechanism. The masseter muscle was then incised vertically to expose the bony fragments. Skeletal traction was applied using a 2.0 mm × 8.0 mm screw inserted into the mandibular angle, with a percutaneous wire used to mobilize the distal fragment. After adequate access was obtained, reduction and osteosynthesis with the most suitable 2.0 titanium plates were performed (Figure 2a). The wound was closed in layers: muscle (2/0 absorbable stitches), periauricular fascia, and subcutaneous tissue with absorbable 4/0 stitches, and skin with non-absorbable 5/0 sutures. A compressive preauricular dressing application was finished the procedure.

For patients treated with endoscope-assisted ORIF, an intraoral approach was employed using the Storz system. Prior to incision, the intraoral mucosa and perioral skin (lips and cheeks) were disinfected with a non-alcoholic antiseptic solution. Local infiltration with vasoconstrictor (adrenaline 1:200,000–1:100,000) was performed, and after approximately 15 min, once the vasoconstrictive effect was achieved, an intraoral incision was made. The incision extended along the anterior border of the mandibular ramus (Figure 1b), then inferiorly and posteriorly along the external oblique ridge, parallel to the gingival margin, reaching the first molar region. The periosteum was elevated endoscopically along the ramus to the coronoid base and fracture line from lateral aspect, with dissection kept in close contact with bone to avoid masseter injury. Skeletal traction was applied using a 2.0 mm × 8.0 mm screw inserted into the mandibular body, with a percutaneous wire used to mobilize the distal fragment. Fracture reduction was achieved under endoscopic control, and a transbuccal trocar was introduced through a small preauricular skin incision following saline infiltration to protect the facial nerve branches and create a channel for the trocar. A 2.0 titanium miniplate with built-in clip for trocar were used. The plates were positioned intraorally and fixed with sequential screw placement (Figure 2b), ensuring accurate alignment and stable occlusion. After hemostasis, the trocar was removed, intraoral wounds were closed with two layers of resorbable 3/0 stitches, and the skin wound was sutured with one or two interrupted non-absorbable 5/0 sutures, and a compressive preauricular dressing was applied.

Endoscopic assistance allowed magnification of the operative field, precise fracture management, and fixation with minimal disruption of surrounding tissues [48]. High-quality imaging was ensured by proper camera white-balance adjustment and verification of intraoperative sharpness. The optical system consisted of an HD (or higher) endoscopic camera connected to a light-guide cable. A 4 mm, 18 cm, 30° wide-angle telescope served as the standard optic. To protect the optics during surgery, the telescope was fitted with an endoscopic sheath with an integrated optical preparator. The sheath’s distal end, shaped like a bent raspatory with a wide fenestration, facilitated tissue elevation, fragment repositioning, and miniplate stabilization, while keeping the cheek trocar centered in the operative view.

In both cases, the fixing material was chosen from straight plates, ACP plates, and XCP plates (ChM, Juchnowiec Kościelny, Poland, www.chm.eu accessed on 2 September 2025) [49,50].

Immediately after surgery, patients underwent blood tests and assessment of facial nerve function using the six-grade House–Brackmann scale. Postoperative evaluation also included wound observation and computed tomography (CT). Follow-up consisted of repeated assessment of facial nerve function with the House–Brackmann scale and evaluation of temporomandibular joint (TMJ) function using the four-grade Helkimo Index.

The collected data were anonymized, and statistical analyses were performed using Statgraphics Centurion 18 (Statgraphics Technologies Inc., The Plains, Warrenton, VA, USA; www.statgraphics.com, accessed on 10 August 2025). The analysis included tests of normality, Student’s *t*-test for mean comparison, and ANOVA or the Kruskal–Wallis test to evaluate the influence of the surgical approach. A *p*-value < 0.05 was considered statistically significant.

## 3. Results

This retrospective observational study initially screened 182 medical records of patients who had undergone surgical treatment for mandibular condylar fractures. After a detailed review against the predefined inclusion and exclusion criteria, 49 cases were eligible and included in the final analysis. Patients were divided into two treatment groups: one managed with an endoscope-assisted intraoral approach and the other treated with ORIF via a retromandibular transparotid approach. In the intraoral group, there were 6 females and 12 males (mean age 37 ± 17 years), while the retromandibular group consisted of 7 females and 24 males (mean age 36 ± 15 years). Comparative analysis of baseline demographics showed no statistically significant differences between the groups with respect to sex distribution (χ^2^ test of independence) or age (Kruskal–Wallis test). Similarly, place of residence (urban/rural; intraoral 15:3, retromandibular 23:8), BMI, and the presence of internal comorbidities (0.4 ± 0.8 vs. 0.2 ± 0.5) did not differ significantly between groups. These findings confirmed that both cohorts were demographically and clinically comparable at baseline, ensuring the validity of subsequent outcome comparisons (Table 1).

Moreover, the two groups were comparable in terms of injury characteristics. These included the number of basal and low-neck condylar fractures (16:2 vs. 28:3, respectively), the distribution of unilateral and bilateral fractures, causes of injury (Assault/Fall/Sports/Vehicle/Workplace, 9:3:1:4:1 vs. 12:11:2:6:0, respectively), and the use of narcotic substances at the time of injury (No/Yes, 8:10 vs. 16:15, respectively). No statistically significant differences were observed (Table 2).

**Figure 3:**
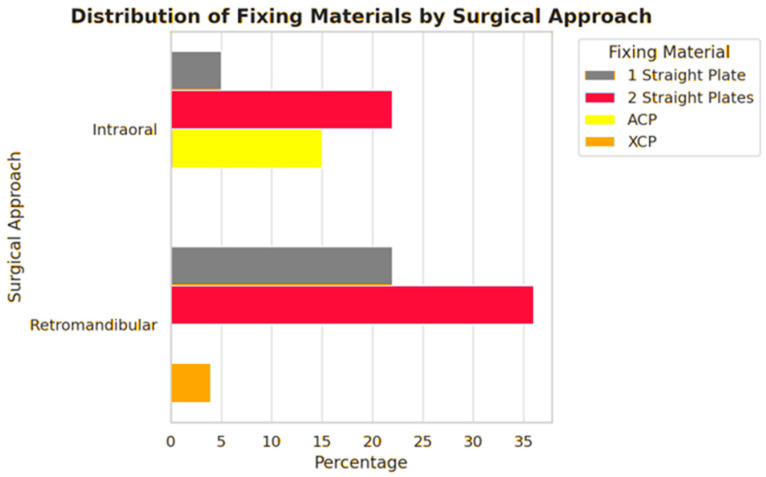
The choice of fixation material in endoscopic and conventional ORIF surgeries. Straight plates were more frequently used in endoscopic procedures. The vertical axis represents the surgical approach (intraoral for endoscopic surgeries and retromandibular for conventional ORIF surgeries), while the horizontal axis shows the proportion of patients treated with a given fixation material (in %).

Analysis of surgical data revealed a significant difference between the endoscopic and retromandibular groups with respect to the fixation material used (*p* < 0.05). In endoscope-assisted procedures, surgeons more frequently selected straight plates (one or two) rather than dedicated prefabricated plates (ACP or XCP) (Figure 3, Table 3).

**Figure 4:**
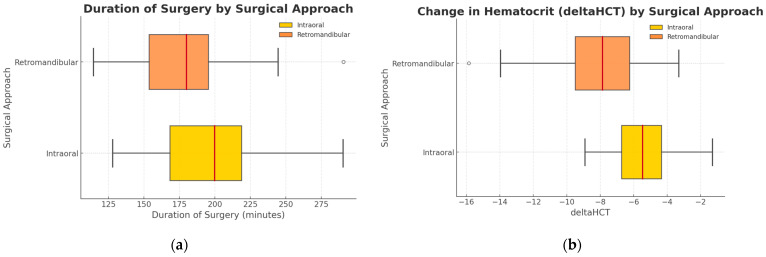
Overview of surgery duration and postsurgical hematocrit values; (**a**) Visualization of surgery duration for two techniques: endoscopic intraoral approach and ORIF with retromandibular approach. The vertical axis represents the type of surgical approach, while the horizontal axis shows surgery duration in minutes (*p* > 0.05). (**b**) In both groups compared, the hematocrit value decreased on the day of discharge from the hospital. This is an adverse effect of the surgical procedure performed. The vertical axis represents the type of surgical approach, while the horizontal axis shows the alteration in hematocrit value between the time of admission to and discharge from hospital (percentage points) (*p* < 0.05). The lower value means a worse result.

In terms of the duration of surgery, although the study suggested that surgery with endoscopic assistance takes longer on average (intraoral approach/retromandibular approach median 165 and IQR 65–340 vs. median 135 and IQR 70–375), but the statistics for this variable were underpowered (*p* > 0.05) and the observation should not be interpreted as significant or reliable (Table 3). Despite the slightly longer procedures with endoscope assistance (198 ± 85 min. vs. 168 ± 75 min.), the decrease in hematocrit values was significantly less (−3.8 ± 3.1 percentage points) than in patients with the retromandibular approach (−7.8 ± 4.0 percentage points), where the statistical significance was reached (*p* = 0.0006) (Figure 4).

**Figure 5:**
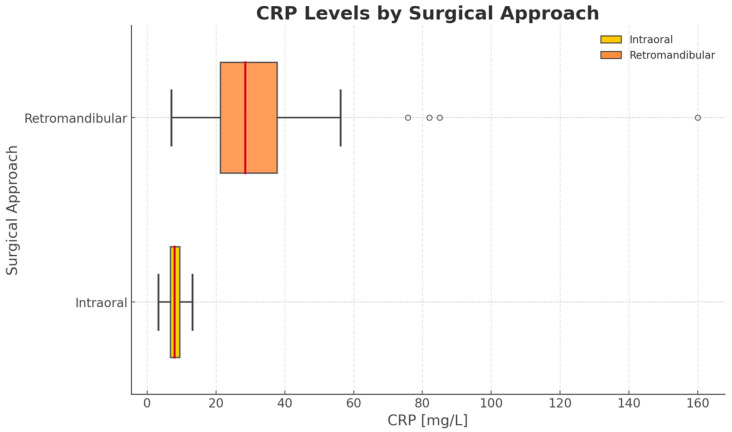
Relation of the post-operational CRP levels and surgical approaches (intraoral vs. retromandibular). The intraoral approach used during endoscopic treatment is associated with lower CRP levels. The vertical axis represents the type of surgical approach with corresponding techniques (intraoral approach + endoscopic surgery, retromandibular approach + traditional ORIF). The horizontal axis shows CRP levels (mg/L) (*p* = 0.046).

The study revealed that, with regard to postoperative outcomes, there could be a potential relation between the surgical approach and the postoperative CRP level (Figure 5). The study indicates that patients treated with an endoscopic technique via the intraoral approach had lower postoperative CRP levels than patients treated with ORIF via the retromandibular approach (intraoral/retromandibular median 1.75 and IQR 0.5–86.3 vs. median 7.2 and IQR 0.3–158.9) (Table 4). This observation was statistically significant (*p* < 0.05).

The results of the Helkimo Index, which assesses TMJ function, and the House–Brackmann Scale, which examines facial nerve function, did not indicate significant differences between the endoscopic and ORIF groups. No statistical differences were detected in either examination or treatment method. Therefore, one may conclude that there is no relation between the surgical technique used to treat basal or low-neck fractures and the postoperative function of TMJ and postoperative condition of facial nerve and its recovery process (Table 4).

Considering the relation of surgical technique with postoperative complications, there was a relationship between endoscope-assisted condylar surgery and the risk of impaired bone healing after surgery. This manifested as a higher rate of non-union of fractured mandibular condylar process fragments compared with the traditional ORIF group. This relation reached statistical significance (*p* < 0.05). However, this factor did not result in a higher rate of reoperations for mandibular condylar process fractures (Table 4).

To further analyze the two cases of fracture non-union in the endoscopic group, more detailed data are provided. Both patients were women and the fixation was ACP plate. The first was 46 years old, with a low-neck fracture and no comorbidities. Immediate post-operative CT examination confirmed proper fracture fixation; however, at the 1-month follow-up visit, translocation of bone fragments with non-union was detected, and a reoperation was performed. The second case was a 77-year-old woman with a basal fracture. She presented with comorbidities, including hypertension and chronic obstructive pulmonary disease, and was undergoing diagnostic evaluation for polyneuropathy. CT scans of the maxillofacial bones revealed low bone density, and the patient was referred for bone densitometry. Non-union was diagnosed three months after surgery.

## 4. Discussion

The current literature reports numerous references to endoscope-assisted treatment in maxillofacial surgery, a technique that is continually refined and increasingly adopted in many centers [51,52,53]. While its advantages are well recognized, it remains technically demanding and requires substantial surgical experience to ensure safety and effectiveness. Several studies emphasize the importance of training: Foletti et al. highlighted the learning curve in condylar fracture management, showing skill improvement after animal model practice [51]; Mannion and Loukota reported that over a quarter of surgeons required professional training [53]; and Aziz et al. demonstrated that inexperienced surgeons needed two to three times longer to perform procedures compared with experienced colleagues [54]. Increased experience is consistently associated with fewer post-operative complications [55], and differences in expertise across centers may explain the variability in clinical outcomes reported in the literature.

Our study and literature review indicate that patient age does not affect the risk of post-operative complications after endoscope-assisted treatment of condylar process fractures. Sinha and Natarajan found no significant differences related to age or gender between intraoral and extraoral approaches [56], while Neuhaus et al. likewise reported no correlation between gender and post-operative outcomes [4]. In contrast, several studies on traditional ORIF identified female gender as a risk factor for post-operative facial nerve palsy [16,57,58]. Research on maxillofacial trauma further suggests that patients from urban areas are more prone to injury than those from rural settings, with injury patterns differing by residence [59,60,61,62]. However, evidence linking endoscopic techniques to postoperative outcomes remains limited, and our findings support the view that a patient’s place of residence does not significantly affect complication rates or recovery [63].

Several studies have suggested a lower risk of facial nerve injury with the endoscopic approach compared with traditional ORIF [40,64,65]. However, other reports and a meta-analysis by Cavalcanti et al. found no significant differences in postoperative outcomes [66,67]. Our results are in line with these latter findings.

TMJ function, assessed using the Helkimo Index, was comparable between intraoral and extraoral groups, consistent with previous studies reporting no significant differences between endoscopic and extraoral ORIF in subcondylar fractures [40,56,64,68].

The minimally invasive nature of endoscope-assisted surgery was reflected in certain postoperative outcomes. Hematocrit (HCT), which indicates blood loss and hydration status and helps determine transfusion needs and circulatory stability [69,70], was significantly higher in the intraoral group compared with the extraoral group. This may be attributed to reduced tissue trauma, smaller incisions, and decreased vascular injury associated with endoscopic surgery. Another marker of tissue injury and invasiveness is the postoperative CRP level [71]. Elevated CRP after trauma or surgery is considered a physiological response, particularly within the first 24–48 h [72,73,74]. In our study, CRP levels were lower in the endoscopic group than in patients treated with traditional ORIF, which is partially consistent with the findings of Gwak et al., who reported higher CRP concentrations in patients with greater tissue injury and more invasive maxillofacial procedures [75].

Several studies have reported that endoscope-assisted management may require longer operative times than conventional ORIF, as shown in a meta-analysis by Bera et al. [76] and some clinical trials [40,56,77]. However, other studies indicated that procedures can be significantly shorter [78], particularly when performed by experienced surgeons [51,79]. Operative duration is also closely related to fracture complexity [80], and this technique is not suitable for high-neck or mandibular head fractures. Patient selection may be optimized using the Warsaw Complexity Scale [81]. In our study, mean operative times were 198 min for the endoscopic group and 168 min for the ORIF group, a difference that did not reach statistical significance.

Despite its minimally invasive nature, the intraoral endoscopic approach is associated with disadvantages, including restricted visualization and limited ability to manipulate bone fragments compared with the extraoral approach. Moreover, in the event of intraoperative bleeding from vessels in this region—most commonly the retromandibular vein—the endoscopic procedure becomes more challenging to control, and hemostasis may be more difficult to achieve [56,82]. These challenges may partly explain the two cases of non-union observed in our study. In the 46-year-old patient, the most probable reason was a technical factor—failure of fixation due to screw loosening, which likely prevented rigid stabilization; additionally, the treatment of the fracture was technically difficult because it was a condylar neck fracture. In contrast, in the 77-year-old patient, patient-related factors such as advanced age, general health condition, and a hypothetic polyneuropathy (under diagnostic evaluation) may have influenced the outcome, possibly in association with osteoporosis or another metabolic dysfunction (because of the visible lower bone density). Regarding fixation material, we suppose that plate shape had no influence on the occurrence of the complication, and the presence of the ACP plate in both non-union cases was coincidental.

Dedicated plates have a fixed spacing and arm angulation. If this spacing does not coincide with the tension and compression lines specified by Meyer [83] and Kessler [84], straight plates are used and placed along the ideal osteosynthesis line. Therefore, the use of specific plates should depend on the width of the base of the mandibular condyle [85] and the height of the fracture site.

The wider adoption of endoscope-assisted surgery is limited by the high cost of equipment and dedicated instruments [86,87]. However, the intraoral approach can also be performed without endoscopic assistance, using an angled screwdriver system as an alternative [88,89].

The limitations of the study include the scope of the comparison, which was restricted to basal and low-neck fractures. Another limitation was that two different surgeons performed the operations utilizing the intraoral and retromandibular approaches, which may have introduced operator-related bias. Finally, the study was limited by the relatively small sample size. These findings highlight the need for future randomized studies, ideally with larger cohorts and with both techniques performed by the same operator.

Based on the available literature and our own experience, future research should focus on the development of fixation materials and instruments specifically dedicated to endoscope-assisted management of condylar fractures, as current systems are still more adapted to extraoral approaches [90,91,92,93,94,95,96,97,98]. Clinical and technical studies are also needed to enhance the applicability and reproducibility of endoscopic methods, as the amount of evidence in this field remains limited [99,100,101]. Another important direction is the wider implementation of advanced 3D and virtual planning systems [13,102], which could improve predictability and facilitate surgical procedures. Finally, robotic modalities may also prove useful in this anatomically challenging region [103,104].

## 5. Conclusions

The choice of surgical approach for base and low-neck fractures of the condylar process remains a subject of debate. The intraoral approach is minimally invasive but technically demanding; however, in cases of simple basal fractures it may be relatively straightforward to perform for experienced operators. In contrast, the retromandibular approach is considered a fast, safe, cost-effective, and widely applied method. Overall, both techniques can be regarded as safe treatment options, and the choice should depend on the fracture pattern, the surgeon’s experience, and the specific clinical context.

## Figures and Tables

**Table 1:** Overview of patients’ demographics characteristics. The groups compared in the study were similar.

Variable	Intraoral Approach (n = 18)	Retromandibular Approach (n = 31)	*p*-Value
Age (mean ± SD) [years]	37 ± 17	36 ± 15	*p* = 0.983
Gender			*p* = 0.627
Female	6	7	
Male	12	24	
Patient residence			*p* = 0.701
Rural	3	8	
Urban	15	23	
BMI (mean ± SD) [kg/m^2^]	23.17 ± 3.8	23.05 ± 4.4	*p* = 0.993
Comorbidities			*p* = 0.095
3 diseases	1	0	
2 diseases	0	1	
1 disease	5	3	
Healthy Generally	12	27	

SD—Standard Deviation; The results are not statistically significant (*p* > 0.05).

**Table 2:** Injury characteristics in investigated patients.

Variable	Intraoral Approach (n = 18)	Retromandibular Approach (n = 31)	*p*-Value
Type of condylar fracture			*p* = 0.580
Basal	16	28	
Low-neck	2	3	
Number of injured condyles			*p* = 0.890
Unilateral	15	24	
Bilateral	3	7	
Reason of injury			*p* = 0.473
Assault	9	12	
Fall	3	11	
Sports	1	2	
Vehicle	4	6	
Workplace	1	0	
Use of narcotics substances during injury			*p* = 0.628
No	8	16	
Yes	10	15	

The results are not statistically significant (*p* > 0.05).

**Table 3:** Overview of surgical data.

Variable	Intraoral Approach (n = 18)	Retromandibular Approach (n = 31)	*p*-Value
Duration of surgery (mean ±SD) [minutes]	198 ± 85	168 ± 75	*p* = 0.198
Type of fixing material			***p =* 0.027**
1 straight plate	2	0	
2 straight plates	10	12	
ACP plate	6	17	
XCP plate	0	2	

SD—Standard Deviation; bolded values represent results that were noted as statistically significant (*p* < 0.05).

**Table 4:** Overview on the postoperative data and complications rate.

**Variable**	**Intraoral Approach** **(n = 18)**	**Retromandibular** **Approach** **(n = 31)**	***p*-Value**
CRP (median and IQR) [mg/L]	1.75 (0.5–86.3)	7.2 (0.3–158.9)	***p* = 0.046**
Helkimo Index 6M after surgery			*p* = 0.386
0	10	21	
I	6	1	
II	1	9	
III	1	0	
House-Brackmann Scale			
0M after surgery			*p* = 0.238
I	13	26	
II	0	2	
III	2	2	
IV	2	1	
V	1	0	
VI	0	0	
6M after surgery			All cases recovered
I	18	31	
II	0	0	
III	0	0	
IV	0	0	
V	0	0	
VI	0	0	
Post-surgical Complications			
Salivary fistula	0	1	
Lingual nerve disfunction	1	0	
Skin paresthesia	3	4	*p* = 1.000
Fracture late union	1	1	*p* = 1.000
Fracture non-union	2	0	
Screw in mandibular Canal	1	0	
No complications	13	29	*p* = 0.102
Reoperation			*p* = 0.252
No	16	31	
Yes	2	0	

0M—immediately after surgery, 6M—6 months after surgery; bolded values represent results that were noted as statistically significant (*p* < 0.05).

## Data Availability

The original data presented in the study are openly available on YouTube at @marcinkozakiewicz5618 and @moki2121 (accessed on 10 August 2025).

## Abbreviations

The following abbreviations are used in this manuscript:

ORIF	Open Rigid Internal Fixation
TMJ	Temporomandibular Joint
HCT	Hematocrit
CRP	C-reactive Protein Level
BMI	Body Mass Index
